# Typhoid Fever*The editors are sorry that their crowded space prevents this essay from being given in full.

**Published:** 1887-12

**Authors:** J. V. Allen


TYPHOID FEVER* was the subject of an interesting paper, read by Dr. John V. Allen. After reviewing the clinical history, pathology, causation, prophylaxis, etc., of this disease, Dr. Allen gave the foregoing repertory of the therapeutics of anal hæmorrhages, and these hints upon dietetic treatment of typhoid fever:

The homœopathic treatment of this disease consists, as you know, in giving the indicated remedy, and prescribing to the totality of symptoms. Such symptoms as are peculiar to the disease should not be our guide as characteristic of a certain remedy, viz.: nosebleed, subsultus tendinum, dryness of tongue, etc., without each becomes excessive or presents some peculiar feature which is not pathognomonic of the disease.

Another point is the administration of the remedy: after the

* The editors are sorry that their crowded space prevents this essay from being given in full.

simillimum has been found, great care should be used in the repetition of the dose; if after being given improvement commences, do not repeat until improvement ceases, or some unforeseen complications arise, and then with the greatest care, as no other disease is more susceptible to drug action and aggravation than this. * * *

The diet should be selected with great care, all solid food rigidly withheld; nothing should be given as an article of diet which has not its digestion in the stomach, or leaves very little duty for the bowels to perform. Eggs, milk, and arrowroot in various combinations should form the staple articles of food; beef extracts and broths should be given at stated intervals, as they act both as a nutritive and stimulant, and also help to make up for the waste which is constantly going on.

Before closing, I would like to suggest one cow's milk as the special article of diet. When we use milk which has been collected from several cows, we expose our patient, who in this condition is very susceptible to any influence, whether it be in diet or imprudence otherwise. It is as impossible to find several cows which are free from a diseased condition as two or more of the human family are at the same time, and if one of the many cows is sick, so will the milk of the many be contaminated, and our patient rendered liable to be more or less severely affected thereby.

I think it important that one healthy cow should be selected, and the milk of morning and evening served to the patient. Care must be taken to see that she is fed exclusively upon bran-meal and hay, and not upon garbage or decayed vegetable matter, like most of our dairy cows are fed. I think it very essential that this matter should be carefully looked into, as it is a duty which the physician owes his patient, and one of no trivial matter.

